# Circadian and Sleep Dysfunctions in Neurodegenerative Disorders—An Update

**DOI:** 10.3389/fnins.2020.627330

**Published:** 2021-01-18

**Authors:** Karim Fifel, Aleksandar Videnovic

**Affiliations:** ^1^International Institute for Integrative Sleep Medicine (WPI-IIIS), University of Tsukuba, Tsukuba, Japan; ^2^Movement Disorders Unit and Division of Sleep Medicine, Massachusetts General Hospital and Harvard Medical School, Boston, MA, United States

**Keywords:** Alzheimer, Parkinson, Huntington, sleep, circadian system

## Abstract

Disruptions of sleep and circadian rhythms are among the most debilitating symptoms in patients with neurodegenerative diseases. Their underlying pathophysiology is multilayered and multifactorial. Recent evidence suggests that sleep and circadian disturbances may influence the neurodegenerative processes as well as be their consequence. In this perspective, we provide an update of the current understanding of sleep and circadian dysregulation in Alzheimer’s, Parkinson’s, and Huntington’s diseases.

## Introduction

Neurodegenerative disorders are characterized by a constellation of multiple impairments in diverse functional domains (Jucker and Walker, 2013). In addition to the primary symptoms characterizing each neurodegenerative disease [i.e., memory dysfunction in Alzheimer’s disease (AD) and motor disorders in Parkinson’s disease (PD), and Huntington’s disease (HD)], several physiological functions deteriorate over disease progression and will ultimately dominate the clinical picture at advanced stages of the diseases (Bates et al., 2015; Masters et al., 2015; Poewe et al., 2017; Long and Holtzman, 2019). The primary neurodegenerative processes affect regions involved in the regulation of sleep, alertness and circadian rhythms, leading to disrupted sleep-wake cycles and circadian dysregulation (Coogan et al., 2013; Videnovic and Golombek, 2013, 2017; Videnovic et al., 2014a,b; Mattis and Sehgal, 2016; Musiek and Holtzman, 2016; Videnovic and Willis, 2016; De Pablo-Fernández et al., 2017; Fifel, 2017; La Morgia et al., 2017; Li et al., 2017; De Lazzari et al., 2018; Leng et al., 2019; Duncan, 2020). Increasing evidence points to deleterious effects of disrupted sleep and circadian homeostasis on the biological processes that underlie neurodegeneration. This bi-directional relationship between neurodegeneration and sleep and circadian systems opens immense opportunities for the development of novel treatment approaches for these disorders (Musiek and Holtzman, 2016; Leng et al., 2019). In this perspective, we summarize the symptomatology of sleep and circadian rhythms in neurodegenerative disorders, with an emphasis on the most common neurodegenerative disorders—AD, PD, and HD. Mechanistic insights mediating sleep and circadian dysfunction in these neurodegenerative diseases are highlighted as well. We have focused on human clinical investigations with occasional referral to animal studies for mechanistic insights.

## Circadian Dysfunction in Neurodegenerative Disorders

Circadian organization of biological processes is manifest, and plays a paramount role, at every level of organization from gene expression and cellular redox state regulation to intra- and inter-organ physiological coordination (Bass and Lazar, 2016; Patke et al., 2020). Given this complex and hierarchical organization of circadian biology, it is not a surprise that chronic breakdown of circadian rhythms is a significant risk factor for a range of diseases, including cancer, metabolic disorders, psychiatric disturbances, and neurodegenerative diseases (Bass and Lazar, 2016; Patke et al., 2020). In neurodegenerative disorders, three types of circadian alterations can be defined: behavioral, physiological and molecular alterations.

The most evident and extensively studied overt behavioral circadian disturbance in patients with neurodegenerative diseases is the alteration of sleep/wake behavior. Given the complex and widespread neuronal centers governing the quality and quantity of sleep/wake cycle (Saper et al., 2005) and the widespread nature of neurodegeneration and neural dysfunction in patients with neurodegenerative disorders (Jucker and Walker, 2013), sleep disruption usually precedes respective clinical diagnoses by several years and contribute significantly to the deterioration of the patients’ quality of life. Some of the sleep alterations in neurodegenerative diseases are exaggerated forms of age-related sleep alterations (Mander et al., 2017). A typical example of this in AD, PD, and HD patients is the progressive fragmentation of sleep cycle over time. A more detailed discussion of sleep disorders in AD, PD, and HD diseases will follow in the next section. Other studies have capitalized on actigraphy to continuously monitor the progression of rest/activity rhythms in community-dwelling patients with neurodegenerative diseases (Leng et al., 2019). These studies have shown several alterations including frequent daytime naps, altered phase angles as evident by shifts in both bedtime and wake times, rest/activity fragmentation, and reduced amplitudes (Ortiz-Tudela et al., 2014; Hooghiemstra et al., 2015; Wang et al., 2015; Weissova et al., 2016). Interestingly, four recent studies have revealed robust association between weakened rest/activity rhythmicity and the subsequent development of AD and PD years later (Musiek et al., 2018; Lysen et al., 2019; Leng et al., 2020; Li et al., 2020). Circadian disturbances could therefore act as independent risk factor for the development of neurodegenerative diseases. Future studies should investigate the mechanisms behind these associations. Further, circadian rhythms could be a promising therapeutic target for the prevention and management of neurodegenerative diseases.

Alterations of additional physiological processes that are under the regulation of the circadian pacemaker, the suprachiasmatic nucleus (SCN) have been reported in these neurodegenerative disorders (Fifel, 2017; Fifel and DeBoer, 2020; Fifel and Videnovic, 2020). Several investigations in AD, PD and HD patients have revealed alterations of the circadian amplitude of cortisol rhythm (Hartmann et al., 1997; Hatfield et al., 2004; Aziz et al., 2009a; Adamczak-Ratajczak et al., 2017), reversal or complete loss of nychthemeral modulation of blood pressure and heart rate variability (Ejaz et al., 2006; Chen et al., 2013; Vetrano et al., 2015; Diago et al., 2018; Baschieri and Cortelli, 2019), phase desynchrony and amplitude disruption of melatonin-release cycle (Skene and Swaab, 2003; Aziz et al., 2009b; Kalliolia et al., 2014; Adamczak-Ratajczak et al., 2017; Fifel, 2017), dampening or reversal of the diurnal pattern of urine excretion (Hineno et al., 1994; Ouslander et al., 1998) and impaired body temperature (Raupach et al., 2019). Collectively these alterations demonstrate that patients with neurodegenerative diseases exhibit a global circadian desynchrony. This state of systemic desynchrony is known to precipitate deleterious impact on global health that is worse than having a dysfunctional SCN clock (Fernandez et al., 2014) and is therefore expected to causally contribute to health impairment in patients with neurodegenerative diseases.

The additional body of evidence linking circadian disturbances with neurodegenerative disorders comes from studies investigating circadian molecular markers in patients with neurodegenerative diseases. In PD patients, although all clock genes were not affected, significant alterations of the timing and amplitude of core clock genes such as Bmal1, Bmal2, Per2, and Rev-erbα were reported (Cai et al., 2010; Breen et al., 2014). Breen et al. reported a loss of the circadian expression of Bmal1 in peripheral mononuclear cells of PD patients (Breen et al., 2014). Few studies have also examined the contribution of circadian dysfunction to health impairments in PD patients by exploring the associations between single-nucleotide polymorphism of several clock-related genes and motor and non-motor symptoms of PD (Ding et al., 2011; Hua et al., 2012; Gu et al., 2015; Lou et al., 2018; Mao et al., 2018). Polymorphisms in clock-related genes such as ARNTL, thyrotroph embryonic factor gene, Clock, were proposed as independent risk factors for motor, depression-related and sleep disorders in PD (Gu et al., 2015). These studies suggest that the molecular mechanisms of the clock in peripheral organs of PD patients are altered and could contribute to the behavioral and physiological desynchronization observed in patients with PD (Fifel, 2017). Similar studies centered on clock genes expression in AD and HD are scant. However, few pathological studies using post-mortem brain tissue have shown a loss of the diurnal profile of clock genes (Bmal1, Cry1, Per1) in the pineal gland of patients with preclinical and manifest AD (Wu et al., 2006). Similarly, by examining the expression of clock genes per1, per2 and bmal1 in several brain structures, Cermakian et al. (2011) reported a dyssynchronous expression of several clock genes in different brain structures. In addition to altered clock gene expression, the expression of key SCN neuropeptides such as vasopressin, AVP and VIP, is significantly decreased in patients with AD and HD (Zhou et al., 1995; Liu et al., 2000; Wu et al., 2007; van Wamelen et al., 2013). Collectively, the behavioral, physiological and molecular alterations of the circadian system strongly implicate their involvement as a significant causal factor in the impairment of the quality of life in patients with neurodegenerative diseases.

Similar to the underlying mechanisms of sleep disturbances, the mechanisms of circadian alterations are also multifactorial involving the interactions between several intrinsic neurodegenerative processes (i.e., inflammation, oxidative stress) as well as a dysfunctional communication between central and peripheral clocks (Musiek and Holtzman, 2016; Chauhan et al., 2017). Recently, several molecular pathways linking multiple neurodegenerative processes with the genetic machinery of the circadian clock have been identified (Musiek and Holtzman, 2016; Sharma et al., 2020). However, translational insights from these mechanistic studies aimed at restoring a robust, yet flexible, circadian rhythms in patients with neurodegenerative disorders have not yet materialized.

From a neural network perspective, circadian rhythms are generated by a well-coordinated communication between peripheral clocks and the central clock in the SCN. This is achieved by both neural connections and humoral signals (Hastings et al., 2003; Bass and Lazar, 2016). In neurodegenerative diseases, significant impairments in different parts of this circadian neuronal network have been documented and have been recently reviewed elsewhere (Chauhan et al., 2017; Fifel, 2017; Fifel and DeBoer, 2020; Fifel and Videnovic, 2020). This knowledge is of a paramount importance for future attempts to develop specific network-based therapies for circadian and sleep symptoms in neurodegenerative diseases.

## Sleep Dysfunction in Neurodegenerative Disorders

Disturbances of sleep and alertness are common in neurodegenerative disorders such as Alzheimer’s, Parkinson’s, and Huntington’s diseases. Despite its common presence, sleep disruption is frequently under-reported by patients and their caregivers and under-diagnosed by healthcare professionals. Disrupted sleep negatively affects the quality of life and may impact the safety of patients. Increasingly manifested as these disorders progress, sleep dysfunctions may also predate their onset by years, even decades. For example, REM Sleep Behavior Disorder represents the most significant prodromal manifestation of a synuclein-specific neurodegenerative disorder, and excessive daytime sleepiness has been linked with the development of Parkinson’s disease (Abbott et al., 2005; Hogl et al., 2018).

### Alzheimer’s Disease

Alzheimer’s disease (AD), the most common neurodegenerative disorder, has been associated with sleep and circadian disturbances for a long time. Recent evidence strongly suggests a bi-directional relationship between AD and sleep/circadian disruption (Wang and Holtzman, 2020). Sleep changes that accompany aging are accentuated among individuals affected by AD. In AD, sleep becomes fragmented leading to shorter total sleep time, excessive sleepiness may develop with subsequent frequent napping, and sundowning is often associated with behavioral changes and agitation. These changes affect not only patients but may cause a significant strain on their caregivers (Terum et al., 2017). Alterations in sleep architecture in AD encompass less frequent and poorly formed spindles and K complexes, reduced slow-wave sleep, and changes in the spectral properties of REM sleep (Vitiello et al., 1990; Petit et al., 2004; Guarnieri et al., 2020). Recently the contribution of Obstructive sleep apnea to cognitive impairments in AD patients has been recognized (Bubu et al., 2020; Liguori et al., 2020). Burgeoning evidence demonstrates an increased risk of developing AD in individuals with a long-standing history of disturbed sleep (Virta et al., 2013; Pase et al., 2017). Several recently published meta-analyses reported that individuals with sleep problems had up to 4 times higher risk of AD, cognitive impairment, and preclinical AD (Bubu et al., 2017; Shi et al., 2018). Advance in biomarkers of AD have allowed more sophisticated analysis of the relationship between sleep and AD. Disruption of slow-wave sleep is associated with increased levels of beta-amyloid, whose deposition may impair slow-wave sleep (Ju et al., 2017). Tau aggregation and spreading is known to drive AD neurodegeneration (Holth et al., 2019; Long and Holtzman, 2019). Recent investigations both in mice and humans have shown that not only beta-amyloid, but also tau aggregation is regulated by sleep/wake cycle (Holth et al., 2019). More specifically, recent longitudinal studies have identified changes in NREM slow-wave activity, especially at 1–2 Hz, as specific and reliable EEG markers in forecasting the rate of Aβ accumulation over several subsequent years (Lucey et al., 2019; Winer et al., 2020). Changes in REM sleep, such as reduced REM sleep and prolonged REM latency have also been linked with the development of dementia (Pase et al., 2017). Another mechanism implicating sleep in the progression of AD pathology is related to the glymphatic system. This system, which clears the brain of protein waste products, is mostly active during sleep and suppressed during wakefulness (Xie et al., 2013). Clearance of amyloid or tau proteins by the glymphatic system may therefore be reduced on a background of disrupted sleep (Rasmussen et al., 2018; Nedergaard and Goldman, 2020).

Proper assessment of disturbed sleep in AD necessitate that caregivers be interviewed, as patients may not be aware of these sleep disturbances. From an evidence-based perspective, employing good sleep hygiene, and trazodone seem beneficial for improving sleep quality (McCleery et al., 2014). Trazodone may exhibit additional benefits on cognition with long-term use (reviewed in Ferini-Strambi et al., 2020). Several studies that examined the role of melatonin in managing poor sleep associated with AD found no beneficial effects of several sleep metrics (McCleery et al., 2014).

### Parkinson’s Disease

James Parkinson reported on disrupted sleep in his initial clinical depiction of PD in 1817. The impact of disturbed sleep and alertness, however, has only been systematically examined over the past several decades. Excessive daytime sleepiness and/or disrupted nighttime sleep affect the majority of patients during the lifetime of their PD (Chahine et al., 2017). Fragmented sleep is the most prevalent sleep problem in the PD population (Chahine et al., 2017). Up to 80% of PD patients report poor sleep, which has been characterized by lower sleep times and sleep efficiency on polysomnography (Yong et al., 2011). The multifactorial etiology of poor sleep encompasses co-existent sleep and psychiatric disorders, overnight re-emergence of PD symptoms, effects of antiparkinsonian medications on sleep, autonomic dysfunction as well as neurodegeneration of brain regions responsible for the regulation of the sleep-wake cycle.

Primary sleep disorders have some unique features when co-expressed with PD. REM Sleep Behavior Disorder (RBD), a parasomnia characterized by loss of muscle atonia during REM sleep and dream enactment behavior, affects up to 60% of patients with PD (Sixel-Doring et al., 2016). RBD is of special significance for PD as it represents the most significant prodromal manifestation of an evolving PD and other synucleinopathies—dementia with Lewy bodies and multiple system atrophy. Dream enactment behaviors of RBD pose a substantial risk for injuries of patients and their bed partners. Restless Legs Syndrome (RLS) is another primary sleep disorder frequently associated with PD. In contrast to RBD, whether RLS is also a risk factor for subsequent development of PD is still a subject of debate (Alonso-Navarro et al., 2019; You et al., 2019). True prevalence of RLS in PD is not well established as many mimickers of RLS frequently affect PD patients, such as akathisia, rigidity, and leg cramps (You et al., 2019). While both RLS and PD respond favorably to dopaminergic medications and are worsened by the administration of dopamine blocking medications, there are sharp differences from a neuropathological standpoint. Dopaminergic neuronal loss with co-existent increased iron in substantia nigra PD is in contrast to the absence of neuronal loss and reduced iron content in RLS (Ferini-Strambi et al., 2018). Initially reported within the context of parkinsonism during the 1918 Spanish flu pandemic, sleep disordered breathing seem to have similar prevalence in the PD and general populations (Cochen De Cock et al., 2010). Important differentiators include a higher proportion of central and mixed apneas among PD patients, and a lack of strong associations between elevated body mass index and sleep disordered breathing in the PD population (Lajoie et al., 2020). Excessive daytime sleepiness (EDS) became a focus of medical attention in early the 1990s when reports of EDS and “sleep attacks” in association with the use of dopamine agonists were published (Frucht et al., 1999). The prevalence of EDS in pivotal clinical trials of major dopaminergic medications was 15–30%; Treatment with dopamine agonists carries the highest risk of EDS, followed by the combination therapy with levodopa and dopamine agonist, and levodopa monotherapy (extensively reviewed by Chahine et al., 2017). EDS is more prevalent in PD compared with other chronic diseases (Tandberg et al., 1999). While poor alertness and disturbed sleep share causative factors, there are unique mechanisms underlying disturbed alertness in PD. These include loss of hypocretin neurons in the hypothalamus, neurodegeneration of wake-promoting centers and its projections within the brainstem, and circadian disruption discussed in other segments of this article (Fronczek et al., 2007; Fifel and Videnovic, 2020).

In summary, the landscape of disturbed sleep and alertness in PD is quite rich. The diagnosis and treatment approaches for disturbances of sleep and wake cycles have been presented in several well-written reviews (Amara et al., 2017; Loddo et al., 2017). It is worth emphasizing that further work is needed to develop new and optimize existing PD-specific diagnostic tools for disorders of sleep and wake. There is an overwhelming lack of effective, evidence-based treatments of sleep dysfunction in PD (Seppi et al., 2019). This dictates the need for clinical investigations of sleep therapeutics in PD. Sleep may be an important therapeutic target for alleviating both motor and non-motor manifestations of PD. These approaches may also have favorable effects on the disease progression itself.

### Huntington’s Disease

Huntington’s disease (HD) is a progressive neurodegenerative disease characterized by movement deficits, cognitive deterioration, and psychiatric symptoms. Sleep disturbances are common in HD and affect up to 90% of patients. Despite its common occurrence in HD, sleep disorders have not been so thoroughly investigated as in other neurodegenerative disorders (Zhang et al., 2019). Although disturbed sleep in HD correlated with its severity and duration of illness, poor sleep has been reported in the early stages of the disease, even among asymptomatic carriers of the HD mutation (Arnulf et al., 2008; Lazar et al., 2015). Polysomnography in HD reveals fragmented sleep with reduced N3 stage and increased sleep spindle density (Wiegand et al., 1991). REM sleep appears to be reduced in HD, although this finding has been not replicated in all studies (Hansotia et al., 1985; Wiegand et al., 1991). RBD has been reported in HD (Videnovic et al., 2009). Poor sleep may be associated with depression and other neuropsychiatric manifestations of the disease (Videnovic et al., 2009). This emphasizes the need for timely diagnosis and aggressive treatment of disturbed sleep in patients with HD.

Diagnosis of disorders of sleep and wake in the HD population relies on instruments and tests used in general sleep medicine. Complexities of HD that influence sleep patterns in this population necessitate the development of novel assessment methods for sleep and alertness in HD. There is a paucity of therapeutic studies that focused on disturbed sleep in HD. Managing sleep disfunction is further complicated by complex medication regimens that include medications that may negatively impact sleep and/or daytime alertness. Non-pharmacological strategies such as physical exercise may be a promising approach in the management of sleep dysfunction in HD (Mueller et al., 2019).

## Conclusion and Perspectives

Sleep and circadian rhythms are interconnected and all-encompassing physiological systems that affect virtually all biological functions. Unsurprisingly, circadian disruption can precipitate considerable impairments on human health. In neurogenerative diseases, although it is well established that sleep and circadian dysfunctions are both a consequence of and a causal factor of neurodegenerative processes, holistic investigation of clocks, sleep and neurodegeneration is still in its infancy. The extent of neuropathological dysfunctions in sleep and circadian neural centers is still not fully understood. Additionally, the exact role of clock genes in the regulation of cellular function in different brain structures and peripheral organs is unknown. The accelerating progress in this field is holding promising insights into the development of efficient therapies for improving health condition in patients with neurodegenerative diseases. Chronotherapies such as the use of timed bright light therapy is a recent example of how fundamental research in this field have yielded positive outcomes in patients with neurodegenerative disorders (Fifel and Videnovic, 2018, 2019).

## Author Contributions

Both authors listed have made a substantial, direct and intellectual contribution to the work, and approved it for publication.

## Conflict of Interest

The authors declare that the research was conducted in the absence of any commercial or financial relationships that could be construed as a potential conflict of interest.
